# Prevalence and associated factors of medical device-related pressure injuries in long-term care facilities: a multicenter cross-sectional study

**DOI:** 10.3389/fmed.2026.1889400

**Published:** 2026-08-27

**Authors:** Suzhen Zhang, Xiuying Zhuo, Mingming Zhang, Jianbing He, Qiuni Cai

**Affiliations:** 1 Hospital Administration Office, Zhongshan Hospital of Xiamen University, School of Medicine, Xiamen University, Xiamen, China; 2 Community Health Service Center of Lianqian Street, Siming District, Xiamen, China; 3 Department of General Surgery, Zhongshan Hospital of Xiamen University, School of Medicine, Xiamen University, Xiamen, China; 4 Department of Thoracic Surgery, Zhongshan Hospital of Xiamen University, School of Medicine, Xiamen University, Xiamen, China

**Keywords:** aged, Associated factors, Cross-sectional study, Long-term care facilities, Medical device-related pressure injury, Pressure Ulcer, Prevalence

## Abstract

**Objective:**

To determine the prevalence of medical device-related pressure injuries (MDRPIs) and identify their associated factors among residents in long-term care facilities.

**Methods:**

A multicenter cross-sectional study was conducted between February and April 2025 across 11 long-term care facilities in Xiamen, China. A total of 380 residents were enrolled. MDRPIs were identified through systematic head-to-toe skin inspection and staged according to the National Pressure Injury Advisory Panel classification. Potential associated factors encompassing demographic characteristics, clinical status, device-related variables, and facility-level features were collected. Independent predictors were identified using multivariable logistic regression.

**Results:**

The overall prevalence of MDRPI was 12.4% (47/380; 95% CI: 9.3%–16.1%). Among the 58 documented lesions, the most common sites were the nasal bridge (34.5%), ears (20.7%), and occiput (17.2%). Nasoenteric/nasogastric tubes accounted for 43.1% of injuries, and Stage 1 injuries were predominant (48.3%). Multivariable analysis identified four independent associated factors: edema (OR=3.49, 95% CI: 1.71–7.13, *p* < 0.001), anemia (OR=2.41, 95% CI: 1.23–4.72, *p* = 0.010), use of two devices (OR=2.48, 95% CI: 1.10–5.59, *p* = 0.028) and three or more devices (OR=5.10, 95% CI: 2.20–11.82, *p* < 0.001) compared with one device, and poorer nutritional status (per 1-point decrease in Mini Nutritional Assessment-Short Form score; OR=1.33, 95% CI: 1.10–1.60, *p* = 0.003).

**Conclusions:**

Approximately one in eight long-term care residents had MDRPIs. Edema, anemia, multiple device use, and poor nutritional status were significant modifiable associated factors. Routine assessment of these factors and targeted prevention strategies are warranted to reduce the burden of device-related injuries in long-term care settings.

## 1 Introduction

Pressure injuries (PIs), also known as pressure ulcers, remain a significant global health concern, particularly among aging populations with limited mobility and multiple comorbidities (1). These injuries not only cause pain, reduced quality of life, and prolonged care episodes, but also impose substantial economic burdens on healthcare systems (2). A specific and increasingly recognized category is medical device-related pressure injuries (MDRPIs), defined as injuries that develop at the skin-device interface as a result of sustained pressure, shear, or friction from diagnostic or therapeutic equipment (3). Current epidemiological studies indicate that MDRPIs account for approximately 34.5% of all pressure injuries in acute care facilities (4); however, data specific to long-term care populations remain strikingly limited. The growing burden of MDRPIs has prompted their inclusion in international prevention guidelines as a distinct and critical entity (5).

Long-term care facilities (LTCFs) represent a setting where residents are at particularly high risk for both PIs and MDRPIs. These residents are often older adults with functional decline, sensory deficits, malnutrition, and multiple chronic conditions that necessitate the prolonged use of medical devices such as oxygen tubing, urinary catheters, nasogastric tubes, splints, and tracheostomy ties (6, 7). Unlike in acute care settings, where device use may be transient, the continuous nature of device application in LTCFs—often lasting weeks to months—creates sustained exposure to pressure and moisture, potentially elevating the risk of device-related skin breakdown (8). Recent evidence further suggests that nursing home residents experience unique risk profiles due to prolonged device usage, limited mobility, and suboptimal staffing ratios (1). Despite this vulnerability, most existing research on MDRPIs has focused on intensive care units (ICUs) and general hospital wards, with a particular emphasis on risk factors in ICU patients (9, 10). This has left a critical knowledge gap regarding the prevalence and associated factors of MDRPIs specifically within the LTCF context.

Several studies have documented the overall prevalence of pressure injuries, with estimates ranging widely from 13.5% to 25.2% depending on population characteristics and measurement methods (11). However, only a limited number of investigations have isolated MDRPIs as a distinct outcome in LTCFs, and these often rely on small sample sizes or do not comprehensively assess device-specific risk factors (12). Factors such as device type, duration of wear, fixation methods, skin microclimate, and staff training levels may all influence MDRPI development, yet their relative contributions in the long-term care population remain poorly understood. Moreover, with the growing complexity of care delivered in LTCFs—including the increasing use of non-invasive ventilation, enteral feeding, and advanced wound care devices—the relevance of MDRPIs is expected to grow (13). The 2019 International Pressure Ulcer/Injury Prevention and Treatment Guidelines have explicitly highlighted device-related injuries as a critical research priority, emphasizing the need for context-specific risk assessment (3).

Understanding the burden and determinants of MDRPIs in LTCFs is essential for designing targeted prevention strategies, staff education programs, and device-related protocols. Cross-sectional designs are particularly suited to estimating prevalence and exploring associations at a single time point, thus providing a snapshot of the current situation and generating hypotheses for future longitudinal work. Accordingly, the aim of this study was to determine the prevalence of medical device-related pressure injuries among residents in long-term care facilities and to identify the factors associated with their occurrence.

## 2 Methods

### 2.1 Study design and setting

This was a multicenter, cross-sectional study conducted between February 2025 and April 2025 in 11 LTCFs located in Xiamen, China. The study followed the Strengthening the Reporting of Observational Studies in Epidemiology (STROBE) cross-sectional checklist (14) (Supplementary Materials S1).

### 2.2 Participants

A stratified cluster sampling method was employed to ensure representativeness across facilities of varying sizes and care levels. LTCFs were first stratified by ownership type (public vs. private) and bed capacity (<100 vs. ≥ 100 beds). Within each stratum, facilities were randomly selected, and all eligible residents were invited to participate.

Inclusion criteria were: 1) age ≥60 years; 2) continuous residence in the facility for ≥3 months; 3) use of at least one medical or assistive device for ≥12 hours/day at the time of assessment. This threshold was established to capture ’sustained’ device application and exclude transient use, aligning with the pathophysiological mechanism of MDRPI which requires prolonged interface pressure (3); and 4) provision of written informed consent by the resident or their legally authorized representative.

Exclusion criteria were: 1) were receiving end-of-life care with an expected survival of ≤48 hours, 2) had pre-existing pressure injuries unrelated to medical devices that would interfere with skin assessment, or 3) had severe skin conditions (e.g., generalized eczema, burns) that precluded reliable skin examination. Written informed consent was obtained from all residents with decision-making capacity; for those lacking capacity, proxy consent was obtained from the legal guardian or next of kin.

### 2.3 Sample size

Sample size calculation was based on the formula for cross-sectional prevalence studies:

$$n=Z^{2}\;\times P(1\text{-}P)/d^{2}$$

Assuming an expected MDRPI prevalence of 12.5% (derived from an unpublished preliminary pilot study conducted by our team in December 2024 across two participating LTCFs, which screened 65 residents and identified an MDRPI prevalence of 12.5% (8/64) among the 64 eligible participants), a 95% confidence level (*Z* = 1.96), a precision of 5% (*d* = 0.05), and a design effect of 1.5 to account for clustering, the minimum required sample size was 342. Anticipating a 10% non-response or missing data rate, the final target sample was set at 374 residents.

### 2.4 Data collection and variables

(1) The primary outcome was the presence of at least one medical device-related pressure injury (MDRPI), defined according to the 2019 International Guideline as a localized injury to the skin and/or underlying tissue resulting from sustained pressure, shear, or friction at the interface between the skin and a diagnostic or therapeutic medical device (3). MDRPIs were identified through a systematic head-to-toe skin inspection and staged using the National Pressure Injury Advisory Panel (NPIAP) classification system (Stage 1, 2, 3, 4, unstageable, and deep tissue injury). For each identified injury, the following were recorded on a standardized form: (a) anatomical location (e.g., ear, nasal bridge, occiput, sacrum, heel); (b) associated device type (categorized as nasoenteric/nasogastric tube, urinary catheter, nasal cannula, oxygen face mask, and other); (c) device material (classified as silicone, metallic, ceramic, or composite). The number of MDRPIs per resident and the proportion of residents with at least one MDRPI were calculated.
(2) Demographic characteristics: Age (years), Sex, Body mass index (BMI, kg/m^2^)
(3) Clinical status: comorbidity burden (Charlson Comorbidity Index), mobility level (Braden Scale mobility subscale), cognitive function (Mini-Mental State Examination), and nutritional status (primarily assessed using the Mini Nutritional Assessment-Short Form, MNA-SF; serum albumin levels were also collected as an exploratory biochemical indicator), Self-care ability (Barthel Index of Activities of Daily Living, ADL), Presence of edema (yes/no), anemia.
(4) Device-related factors: Number of devices, and Device duration (days).

### 2.5 Data collection procedures

Data collection was carried out by a team of four trained research nurses who were not employed by the participating facilities. All nurses underwent a two-day standardized training program that included theoretical instruction on MDRPI identification and staging, practical sessions with photographs, and inter-rater reliability testing. Inter-rater reliability for MDRPI staging, assessed using 30 case photographs, achieved a Fleiss’ kappa of 0.85, indicating excellent agreement. Skin inspections were performed at a single time point for each facility during a four-hour window to minimize between-resident variation. Device-related information was obtained through direct observation and from the nursing records. Demographic and clinical data were abstracted from the residents’ electronic health records.

### 2.6 Statistical analysis

Statistical analyses were performed using SPSS 23.0 Continuous variables were tested for normality using the Shapiro–Wilk test and presented as mean ± standard deviation (SD) or median with interquartile range (IQR). Categorical variables were expressed as frequencies and percentages.

Univariate analyses were conducted to screen potential associated factors of MDRPI. Chi-square tests or Fisher's exact tests were used for categorical variables, while independent t-tests or Mann–Whitney U tests were applied for continuous variables. Variables with a p-value <0.05 in univariate analysis were entered into a multivariable binary logistic regression model using the Enter method. Collinearity diagnostics were performed prior to modeling; variables with a variance inflation factor (VIF) > 5 were excluded to prevent multicollinearity. Results were reported as adjusted odds ratios (OR) with 95% confidence intervals (CI). A two-sided *p*-value <0.05 was considered statistically significant.

### 2.7 Ethical considerations

The study protocol was reviewed and approved by the Institutional Review Board of Zhongshan hospital, Xiamen University (Approval No. 2023-183). Written informed consent was obtained from all participants or their legal representatives. The study complied with the Declaration of Helsinki. All personal identifiers were removed prior to analysis, and data were stored on password-protected servers accessible only to the principal investigator.

## 3 Results

### 3.1 Participant characteristics

**Figure 1:**
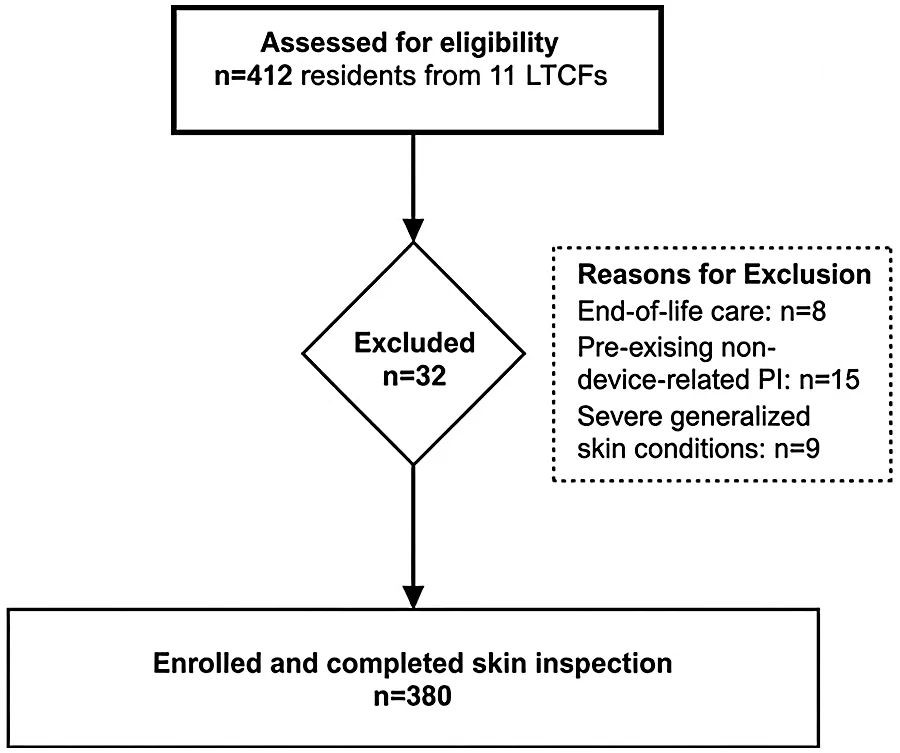
Participant selection flowchart.

A total of 412 residents from 11 LTCFs were initially screened for eligibility. Of these, 32 were excluded due to: end-of-life care (*n* = 8), pre-existing non-device-related pressure injuries interfering with assessment (*n* = 15), or severe generalized skin conditions (*n* = 9). 380 residents were enrolled and completed the skin inspection, yielding a response rate of 100% among eligible participants. The flow of participant selection is shown in Figure 1.

**Figure 2:**
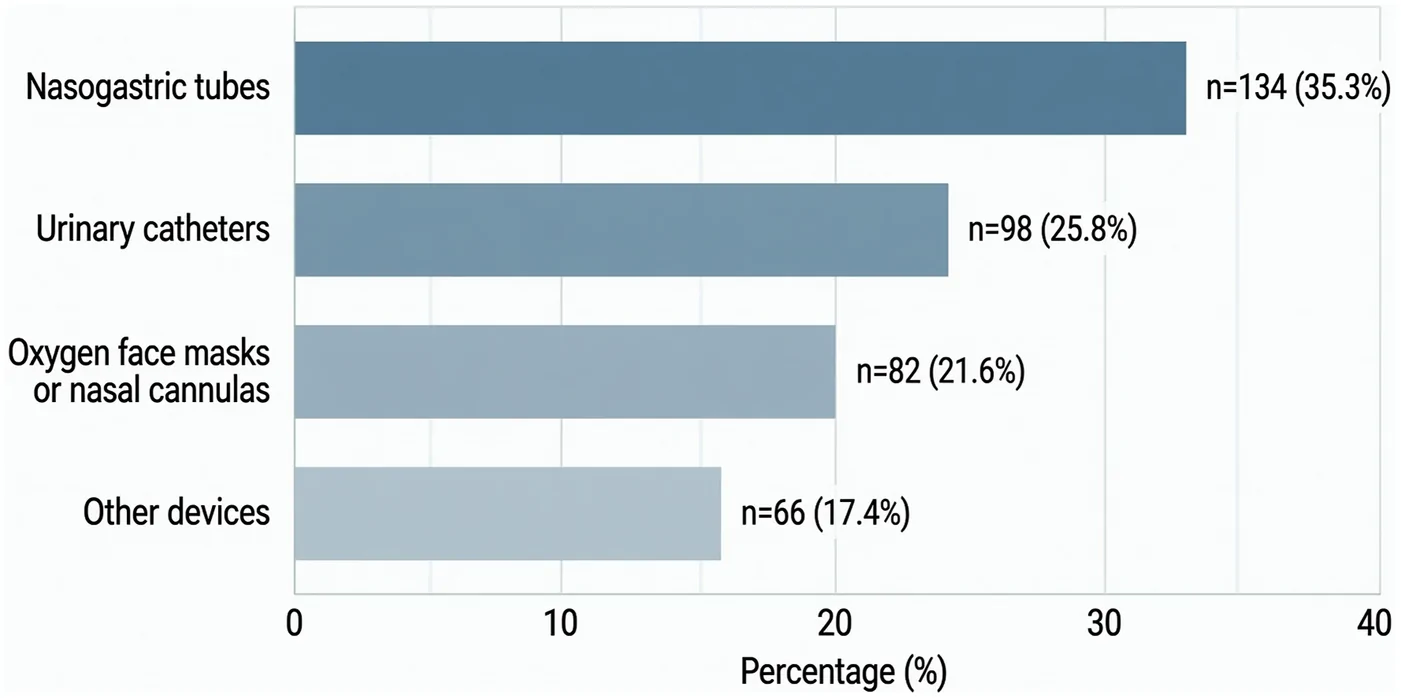
Distribution of medical devices (*n* = 380).

The mean age of participants was 78.4 ± 8.6 years, and 216 (56.8%) were female. A total of 224 residents (58.9%) were from public facilities, and 196 (51.6%) resided in facilities with ≥100 beds. The most common devices were nasogastric tubes (*n* = 134, 35.3%), followed by urinary catheters (*n* = 98, 25.8%), oxygen face masks or nasal cannulas (*n* = 82, 21.6%), and other devices (*n* = 66, 17.4%) (Figure 2). Among participants, 112 (29.5%) had anemia, and 86 (22.6%) presented with edema. Detailed baseline characteristics stratified by MDRPI status are summarized in Table 1.

**Table 1:** Demographic and clinical characteristics of study participants stratified by mDRPI Status (*N* = 380).

Characteristic	Total [ n (%)/median (IQR)/mean ± SD]	MDRPI (*n* = 47)	No MDRPI (*n* = 333)	Test statistic (t/*χ*^2^/Z)	*p*-value
Demographics					
Age (years)	78.4 ± 8.6	82.1 ± 8.0	77.8 ± 8.5	3.30	0.001
Female	216 (56.8)	27 (57.4)	189 (56.8)	0.01	0.928
BMI (kg/m^2^)	22.5 ± 3.8	21.0 ± 3.5	22.7 ± 3.8	2.93	0.004
Clinical status					
Charlson Comorbidity Index	4 (3–6)	5 (4–7)	4 (3–6)	−2.57	0.010
MMSE score	22.1 ± 5.2	20.3 ± 5.5	22.4 ± 5.1	2.55	0.011
MNA-SF score	8 (6–10)	6 (4–8)	8 (7–10)	−4.22	<0.001
Barthel ADL score	45 (25–65)	30 (15–45)	50 (30–70)	−4.15	<0.001
Braden mobility subscale	2 (2–3)	2 (1–2)	2 (2–3)	−4.32	<0.001
Anemia	112 (29.5)	21 (44.7)	91 (27.3)	5.96	0.015
Hypoalbuminemia	69 (18.2)	14 (29.8)	55 (16.5)	4.88	0.027
Edema	86 (22.6)	22 (46.8)	64 (19.2)	17.90	<0.001
Device-related factors					
Number of devices				42.04	<0.001
1	200 (52.6)	8 (17.0)	192 (57.7)		
2	120 (31.6)	18 (38.3)	102 (30.6)		
≥3	60 (15.8)	21 (44.7)	39 (11.7)		
Device duration (days)				9.93	0.007
<30 days	150 (39.5)	10 (21.3)	140 (42.0)		
30-90 days	130 (34.2)	17 (36.2)	113 (33.9)		
>90 days	100 (26.3)	20 (42.6)	80 (24.0)		

SD, standard deviation; IQR, interquartile range; BMI, body mass index; MMSE, mini-mental state examination; MNA-SF, mini nutritional assessment-short form; ADL, activities of daily living. For continuous variables, t-test was used for normally distributed data (mean ± SD) and Mann–Whitney U test for skewed data (median, IQR); Categorical variables were compared using Chi-square test (*χ*^2^).

### 3.2 Prevalence and clinical characteristics of mdrpi

Among the 380 residents assessed, 47 were identified with at least one MDRPI, resulting in an overall prevalence of 12.4% (95% CI: 9.3%–16.1%). A total of 58 distinct MDRPI lesions were documented, with a mean of 1.23 ± 0.52 injuries per affected resident.

The most common anatomical sites for MDRPIs were the nasal bridge/ala (34.5%, *n* = 20), ears (20.7%, *n* = 12), occiput (17.2%, *n* = 10), and heels (13.8%, *n* = 8), with the remaining 13.8% distributed across other sites (e.g., sacrum, chin). Regarding device association, nasoenteric/nasogastric tubes accounted for 43.1% (*n* = 25) of all injuries, followed by nasal cannulae/oxygen face masks (20.7%, *n* = 12), urinary catheters (17.2%, *n* = 10), and other devices (19.0%, *n* = 11). In terms of NPIAP staging, Stage 1 injuries were the most frequent (48.3%, *n* = 28), followed by Stage 2 (37.9%, *n* = 22), Stage 3 (5.2%, *n* = 3), unstageable (5.2%, *n* = 3), and deep tissue injury (3.4%, *n* = 2). No Stage 4 MDRPIs were observed. Device material analysis revealed that silicone-based devices were associated with 60.3% (*n* = 35) of injuries, metallic devices with 25.9% (*n* = 15), and composite materials with 13.8% (*n* = 8). The detailed distribution of MDRPI characteristics is summarized in Table 2.

**Table 2:** Clinical characteristics of medical device-related pressure injuries (*N* = 58 lesions).

Characteristic	n (%)
Anatomical site	
Nasal bridge/ala	20 (34.5)
Ears	12 (20.7)
Occiput	10 (17.2)
Heels	8 (13.8)
Other (sacrum, chin, cheek, etc.)	8 (13.8)
Associated device type	
Nasoenteric/nasogastric tube	25 (43.1)
Nasal cannula/oxygen face mask	12 (20.7)
Urinary catheter	10 (17.2)
Other (splints, tracheostomy ties, etc.)	11 (19.0)
NPIAP stage	
Stage 1	28 (48.3)
Stage 2	22 (37.9)
Stage 3	3 (5.2)
Stage 4	0 (0.0)
Unstageable	3 (5.2)
Deep tissue injury	2 (3.4)
Device material	
Silicone	35 (60.3)
Metallic	15 (25.9)
Composite	8 (13.8)
Ceramic	0 (0.0)

NPIAP, national pressure injury advisory panel. Percentages are based on the total number of lesions (*N* = 58). Percentages may not total 100% due to rounding.

### 3.3 Multivariable logistic regression analysis of factors associated with mdrpi

Univariate analysis (Table 1) identified 12 variables that differed significantly between the MDRPI and non-MDRPI groups (*p* < 0.05): Age, BMI, Charlson Comorbidity Index, MMSE, MNA-SF, Barthel ADL, Braden mobility subscale, anemia, hypoalbuminemia, edema, number of devices, and device duration. These variables were entered into a multivariable logistic regression model.

After adjusting for potential confounders, five factors remained independently associated with MDRPI (Table 3): Edema (OR=3.49, 95% CI: 1.71–7.13, *p* < 0.001); anemia (OR=2.41, 95% CI: 1.23–4.72, *p* = 0.010); number of medical devices: ≥3 vs. 1 (OR=5.10, 95% CI: 2.20–11.82, *p* < 0.001), having 2 devices also increased risk (OR = 2.48, 95% CI: 1.10-5.59, *p* = 0.028); poorer MNA-SF score (per 1-point decrease in MNA-SF score; OR=1.33, 95% CI: 1.10–1.60, *p* = 0.003). Age, BMI, Charlson index, MMSE, Barthel ADL, Braden mobility subscale, device duration and hypoalbuminemia did not remain significant in the final model. The Hosmer–Lemeshow test indicated adequate model fit (*χ*^2^ = 8.42, df = 8, *p* = 0.396), and the Nagelkerke R^2^ was 0.309.

**Table 3:** Multivariable logistic regression analysis of factors independently associated with mDRPI.

Variable	*β* (SE)	Wald χ^2^	OR (95% CI)	p value	VIF
Age	0.01 (0.02)	0.15	1.01 (0.97–1.05)	0.700	1.26
BMI	−0.05 (0.05)	0.93	0.95 (0.86–1.05)	0.335	1.35
Charlson Index	0.06 (0.08)	0.36	1.05 (0.90–1.23)	0.548	2.10
MMSE	0.02 (0.03)	0.40	1.02 (0.96–1.08)	0.527	1.84
Poor nutritional status	0.28 (0.09)	9.68	1.33 (1.10–1.60)	0.003	1.57
Barthel ADL	0.01 (0.01)	0.80	1.01 (0.99–1.03)	0.371	2.42
Braden mobility	0.22 (0.22)	0.93	1.25 (0.79–1.97)	0.335	2.08
Anemia	0.88 (0.34)	6.67	2.41 (1.23–4.72)	0.010	1.22
Hypoalbuminemia	0.30 (0.38)	0.62	1.35 (0.64–2.85)	0.431	1.50
Edema	1.25 (0.36)	12.01	3.49 (1.71–7.13)	<0.001	1.34
Number of devices (ref: 1)					
2	0.91 (0.42)	4.70	2.48 (1.10-5.59)	0.028	2.15
≥3	1.63 (0.43)	14.41	5.10 (2.20-11.82)	<0.001	2.38
Device duration (ref: <30 days)					
30-90 days	0.42 (0.44)	0.93	1.52 (0.65-3.55)	0.334	1.76
>90 days	0.50 (0.42)	1.41	1.65 (0.72-3.78)	0.236	1.89

β, logistic regression coefficient; SE, standard error; OR, odds ratio; CI, confidence interval; VIF, variance inflation factor; MMSE, mini-mental state examination; MNA-SF, mini nutritional assessment-short form, Hosmer–Lemeshow test: χ^2^ = 8.42, df = 8, *p* = 0.396, Nagelkerke R^2^ = 0.309.

## 4 Discussion

This multicenter, cross-sectional study investigated the prevalence and associated factors of medical device-related pressure injuries among residents in long-term care facilities in China. The overall MDRPI prevalence was 12.4%, and four independent associated factors were identified: edema, anemia, higher number of indwelling medical devices, and lower nutritional status (MNA-SF). These findings extend the current understanding of device-related skin injury in the long-term care context, which has been considerably less studied than acute and critical care settings.

The 12.4% prevalence of MDRPI observed in our LTCF cohort is remarkably consistent with the 13.1% prevalence reported by Dang et al. in a large multicenter cross-sectional study of 694 ICU patients across 30 hospitals in China (15), but is substantially higher than the 0.60% overall prevalence reported by Kayser et al. in their analysis of the 2016 International Pressure Ulcer Prevalence (IPUP) survey, which encompassed 99,876 adult patients across US and Canadian acute care, long-term care, rehabilitation, and hospice facilities (16). This similarity is noteworthy given the differences in care acuity and suggests that while the absolute risk may be higher in ICUs, the burden of MDRPI in Chinese LTCFs is comparable to that in some ICU populations, likely reflecting the prolonged device retention times and high proportion of vulnerable older adults in long-term care. Our prevalence also aligns with the pooled estimate of 10% (95% CI: 6–16) from a comprehensive meta-analysis by Jackson et al. (17), and is substantially lower than the 19.3% pooled incidence reported by Zhang et al. in predominantly ICU-based populations (11). The wide variation in reported rates—from 5.01% in a Jordanian ICU (18) to 34% in a Brazilian ICU (19)—as documented by Brophy et al. (20), highlights the profound influence of study design, case mix, and detection protocols. Our finding reinforces that MDRPIs are a significant concern extending well beyond the intensive care unit.

Among the identified associated factors, edema emerged as the strongest independent predictor of MDRPI in our study, conferring a more than threefold increase in risk. The multicenter Chinese study by Dang et al. specifically identified “having skin oedema” as a significant associated factor for MDRPI in adult ICU patients (15). Similarly, a meta-analysis by Gou et al. quantified this risk, reporting that edema was associated with a 3.62-fold increased risk (95% CI: 2.31–5.67) (21). The consistency of this association across acute and long-term care populations strengthens the evidence for edema as a robust, setting-independent risk factor. The underlying mechanism is well established: interstitial fluid accumulation compromises local tissue perfusion, increases the diffusion distance for oxygen and nutrients, and renders the skin more susceptible to deformation and breakdown under sustained external pressure (22). Edema is also frequently a clinical manifestation of hypoalbuminemia and fluid overload, both of which are prevalent in older multimorbid residents. Despite its recognized importance in traditional pressure injury risk, edema has received comparatively less attention in device-specific prevention guidelines; our findings, together with those from ICU-based studies, suggest that routine assessment and management of edema should be a core component of MDRPI prevention bundles across all care settings.

Anemia emerged as another independent risk factor in our analysis, consistent with the meta-analytic findings of Gou et al., who demonstrated that higher hemoglobin levels were protective against MDRPI in ICU patients (21). Hemoglobin is critical for oxygen delivery to skin and subcutaneous tissues subjected to mechanical loading; anemia exacerbates tissue hypoxia and impairs cellular resilience to sustained pressure and shear forces (23). The association between anemia and pressure injury risk has been documented in multiple ICU cohorts (24, 25), and our study extends this evidence to the long-term care population, where anemia is highly prevalent but often underrecognized as a modifiable contributor to skin integrity compromise. Indeed, epidemiological studies have consistently shown that the prevalence of anemia rises substantially with advancing age, exceeding 20% in community-dwelling adults aged 85 years and older (26, 27). Optimization of hemoglobin levels—when clinically indicated and feasible—may represent a supplementary strategy for reducing MDRPI risk in vulnerable older adults.

The number of *in-situ* medical devices was a robust predictor, with a clear dose-response relationship: using two devices more than doubled the risk (OR=2.48), while using three or more devices increased the risk more than fivefold (OR=5.10) compared to using a single device. Each additional device introduces a new skin-device interface and a new site of concentrated pressure, shear, or friction. This finding resonates with the cross-sectional study by Assis et al. in a Brazilian ICU, which reported a 34% MDRPI prevalence and identified multiple specific devices—including nasal catheters, orotracheal tube fixation cords, oximeters, and indwelling urinary catheters—as significantly associated with injury occurrence (19). In our LTCF population, the most commonly implicated devices were nasoenteric/nasogastric tubes and nasal cannulae/oxygen face masks, reflecting the typical care needs of long-term residents. Kaiser et al. also indicated in their study that the most common anatomical sites of MDRPIs occurrence were the ear (29%) and foot (12%). The most closely associated medical devices were nasal oxygen cannulas (26%) and CPAP/BiPAP masks (9%), respectively (16). The predominance of injuries on the nasal bridge and ears in both our study and previous ICU-based investigations (18, 19) underscores that the head and neck region is a universal high-risk zone for device-related pressure damage, regardless of care setting. This pattern highlights the critical importance of regular skin inspection at device-skin interfaces, particularly on the face, and the use of prophylactic dressings and repositioning strategies whenever feasible (23).

Poor nutritional status, as assessed by the MNA-SF, remained independently associated with MDRPI after multivariable adjustment. This finding is consistent with the meta-analysis by Gou et al., which identified lower serum albumin levels as a significant risk factor for MDRPI in ICU patients (21). Malnutrition and unintentional weight loss reduce subcutaneous tissue thickness, diminish the natural cushioning effect against external pressure, and impair wound healing capacity (28). The MNA-SF is a validated, easy-to-administer screening tool specifically designed for geriatric populations, and its predictive utility in our study supports its routine integration into pressure injury risk assessments in LTCFs. Targeted nutritional interventions—including protein-calorie supplementation and micronutrient support—should be considered for residents with low MNA-SF scores, particularly those who require prolonged device use.

In terms of injury characteristics, the predominance of Stage 1 and Stage 2 injuries in our study (86.2% combined) is consistent with the ICU-based findings of Assis et al., where all identified MDRPIs were classified as Stage 1 or Stage 2 (19). This pattern likely reflects the fact that device-related injuries, when detected through systematic surveillance, are frequently identified at an early, reversible stage. However, Erbay Dallı et al. reported that 63.7% of MDRPIs in their cohort were mucosal and therefore unstageable (22), and Saleh and Ibrahim similarly found that 34.2% of injuries involved mucous membranes (18). In our study, unstageable and deep tissue injuries together accounted for 8.6% of lesions, a lower proportion that may partly reflect differences in the types of devices used (e.g., fewer endotracheal tubes in LTCFs) and the absence of systematic mucosal assessment in our skin inspection protocol. The growing recognition of mucosal pressure injuries as a distinct and clinically significant entity (18, 22) suggests that future LTCF-based studies should incorporate explicit mucosal assessment, particularly for residents with nasogastric tubes, urinary catheters, and other devices that contact mucosal surfaces.

### 4.1 Limitation

Several limitations should be acknowledged. First, the cross-sectional design precludes causal inference, and the identified associations should be interpreted as concurrent risk markers rather than causal factors. Second, the study was conducted in a single Chinese city, which may limit the generalisability of the prevalence estimate to regions with different long-term care policies and population profiles. Third, reliance on electronic health records and nursing documentation for certain clinical variables may introduce documentation bias. Fourth, despite excellent inter-rater reliability, some misclassification of deep tissue injury and unstageable injuries cannot be excluded. Fifth, the assessment was restricted to cutaneous pressure injuries; mucosal pressure injuries, which are common with devices such as nasogastric tubes and urinary catheters, were not systematically evaluated, potentially leading to an underestimation of the true MDRPI burden. Sixth, although a multicenter clustered sampling design was employed and a design effect was incorporated into the sample size calculation, standard logistic regression was utilised in the primary analysis; the lack of multilevel modelling to fully account for intra-class correlation at the facility level may lead to slightly underestimated standard errors. Seventh, nutritional status was primarily assessed using the MNA-SF screening tool; serum albumin was collected as an exploratory variable but without concurrent biochemical validation (e.g., total protein, prealbumin), which may limit the precision of nutritional risk stratification. Eighth, we did not collect data on the use of antiplatelet or anticoagulant medications, which could influence local tissue perfusion and microcirculation, thereby potentially modifying susceptibility to pressure-related skin damage at device interfaces. Finally, future studies should employ prospective longitudinal designs, incorporate comprehensive medication histories and a broader panel of nutritional markers, include systematic mucosal and device-care assessments, and apply multilevel analytical approaches to strengthen causal inference and enhance the applicability of preventive protocols.

## 5 Conclusion

This multicenter cross-sectional study demonstrated that medical device-related pressure injuries affected approximately one in eight long-term care residents, highlighting a substantial yet underrecognized burden in this setting. Edema, anemia, malnutrition as assessed by the MNA-SF, and a higher number of indwelling medical devices were independently associated with MDRPI. These clinically accessible and potentially modifiable factors can be routinely assessed in long-term care practice and incorporated into risk-stratified prevention bundles. Future research should employ prospective longitudinal designs to confirm the predictive value of these factors, and evaluate the effectiveness of multifactorial interventions in reducing MDRPI incidence among vulnerable older adults in long-term care facilities.

## Data Availability

The original contributions presented in the study are included in the article/Supplementary Material, further inquiries can be directed to the corresponding author.
